# Patient experience of negative pressure wound therapy: A qualitative study

**DOI:** 10.1002/nop2.1392

**Published:** 2022-10-05

**Authors:** Aiko Miyanaga, Toru Miyanaga, Keiko Sakai, Chizuko Konya, Kimi Asano, Kenichi Shimada

**Affiliations:** ^1^ Department of School of Nursing Kanazawa Medical University Uchinada Japan; ^2^ Department of Plastic Surgery Kanazawa Medical University Uchinada Japan; ^3^ Department of Nursing Ishikawa Prefectural Nursing University Kahoku Japan

**Keywords:** negative pressure wound therapy, patient experience, qualitative study, wound healing

## Abstract

**Aim:**

This study aimed to clarify the treatment experience of patients undergoing negative pressure wound therapy (NPWT).

**Design:**

This study used a qualitative design.

**Methods:**

Seventeen inpatients were semi‐structured interviewed about their experiences of treatment with negative pressure wound therapy.

**Results:**

Inpatients' answers were categorized into seven themes: pain and discomfort associated with treatment, physical limitations owing to attached device, mental burden owing to the odour and noises of the attached device, social limitations owing to the attached device, advances in medical care and science, device personification and mixed feelings towards medical staff. The patients were able to tolerate the aforementioned limitations while feeling attachment and gratitude towards the device created through advances in medical care and science, and towards medical staff who helped them heal. In the future, we plan to develop an NPWT care guide.

## 1 INTRODUCTION

Negative pressure wound therapy (NPWT) is widely used in the treatment of acute and long‐term wounds and wounds after surgery (Zens et al., 2020). It maintains a moist environment, improves blood flow and oxygen supply, delivers nutrients and inducers to the wound surface, removes exudate, promotes granulation, reduces infection and attracts wound margins (Huang et al., 2014). It consists of dressings that cover the wound surface, tubes that apply negative pressure and drain the exudate and canisters (Morykwas et al., 1997). As these items are always worn by the patient, inconveniences during NPWT are expected. Various reports outside of Japan have described the perceptions and experiences of patients who underwent NPWT. Patients' thoughts of receiving NPWT at home focused on the pain associated with the treatment (Abbotts, 2010; Andrews & Upton, 2013; Upton & Andrews, 2013, 2015), limited mobility (Andrews & Upton, 2013), limited social life (Bolas & Holloway, 2012) and dependence on others (Ottosen & Pedersen, 2013). Patients were also inconvenienced by the visibility of the tubes, machine sounds, smell of the exudate and insomnia (Abbotts, 2010; Andrews & Upton, 2013; Upton & Andrews, 2013). Many patients hid the pumps in their coats to reduce the mechanical sounds, whereas others even removed NPWT under certain circumstances (Bolas & Holloway, 2012). In contrast, some patients were satisfied with receiving the latest treatment (Abbotts, 2010). Their desire to return to their normal life encouraged their participation in the treatment (Moffatt et al., 2011); they said that NPWT facilitated wound healing and helped them cope with their inconveniences, thereby improving their lives. Thus, despite the various limitations and inconveniences of NPWT, the aforementioned reports showcased how many patients accepted and were satisfied with the treatment. Abbotts (2010) used a focus group interview technique to efficiently obtain information from patients eligible for NPWT; however, most of the participants were unemployed, and this bias in the target population may not reflect the wishes of employed patients undergoing NPWT. Other studies (Andrews & Upton, 2013; Upton & Andrews, 2013, 2015) conducted surveys of clinicians involved in wound care and patients undergoing treatment to provide a comprehensive overview of the key considerations of NPWT, but did not clarify the patient experience.

In addition, the previous surveys were conducted in Europe and the United States. No existing reports highlight the NPWT experience in Japan. While Japan is highly adapted to inpatients, Europe and the United States primary cater to outpatients. Therefore, the perception of Japanese patients about the inconvenience and treatment of NPWT may differ. In an inpatient setting, clinicians may notice the inconveniences that patients are experiencing owing to NPWT and address them promptly. This study aimed to clarify the treatment experience of hospitalized patients undergoing NPWT.

## 2 METHODS

### 2.1 Design

This was a descriptive qualitative study using a content analysis approach. This design is appropriate for exploring how individuals experienced a phenomenon dependent on their backgrounds, interests and interpretations (Neubauer et al., 2019). Sampling is considered necessary to discover representative or typical exemplars of a broader group for some dimensions of interest and provide the possibility of comparison, or repetition across diverse case types for the dimensions (Polit & Beck, 2017, p. 493). This study used purposive sampling (Liamputtong & Ezzy, 2005). There is no fixed rule for sample size in qualitative research (Gray et al., 2017, pp. 351–352). Therefore, this study formed a sample of 20 participants.

To investigate the perceptions of the hospitalized patients, semi‐structured individual interviews were conducted. The research was conducted and checked according to the COREQ reporting guidelines.

### 2.2 Participants

Patients were admitted for NPWT treatment between June–December 2016 and were selected according to the following inclusion criteria: (1) adults older than 20 years of age, (2) able to independently perform daily living activities and (3) were able to talk about their experiences and cooperate with the researcher. The exclusion criteria included (1) patients who had dementia, (2) those with mental instability or (3) those with motor aphasia. These criteria were adopted to ensure appropriate data provision by the participants.

### 2.3 Data collection

All interviews were conducted by the first author who was not directly involved in patient care. The nurses recruited the participants from the wards where NPWT patients were admitted. During the pre‐interview, the research purpose was explained to the patients. The interview was conducted based on an interview guide (Table 1). The interview began with the question, “What kind of disease or injury did you sustain prior to requiring NPWT treatment?” Next, the participants were asked about their impressions about NPWT, their immediate experiences post‐NPWT, and the resulting difficulties they faced in their daily lives. Furthermore, they were asked about the items they identified as the most difficult to wear while undergoing the NPWT daily. The interview guide was developed after consulting with colleagues who have experience in qualitative research and experts in wound nursing.

**TABLE 1:** Interview guide

Theme	Content
Personal history	What kind of disease or injury did you sustain prior to requiring NPWT treatment?
Machine	What is your impression when attaching or experience immediately after attaching NPWT?
Everyday life	What is the most troublesome thing about undergoing a NPWT treatment during everyday life? How would you describe it?
Treatment	How do you feel about NPWT? Do you have any other thoughts on the treatment?

Abbreviation: NPWT, negative pressure wound therapy.

To ensure the reliability and validity of the interview guide, the content was reviewed by qualitative researchers, wound nursing specialists and plastic surgeons. The interview was conducted in the interview room of the ward. Despite the desirability of multiple interviews with each participant (Seidman, 2006), one interview was deemed sufficient given the brief hospitalization period and the fact the interview only asked about inconveniences in daily life.

Each interview lasted approximately 30 min. Initially, the sample size was set at 20 participants; however, after interviewing 17 participants, no new data emerged. Date “saturation” was achieved when additional participants did not provide any new information, but only duplicated the existing data findings (Polit & Beck, 2017, p. 947). Therefore, it was determined that data saturation had been reached and no further interviews were conducted. All interviews were digitally recorded and transcribed verbatim. The participants' age, sex, underlying disease, aetiology, regions of wounds, and treatment days for NPWT were accessed by their medical records after receiving their consent.

### 2.4 Data analysis

The data were analysed as follows: (1) The transcribed data were meticulously checked to ensure a clear understanding of its overall context. (2) After comparing the extracted codes that were derived from the transcriptions and summarizing their differences and similarities, subthemes were extracted. (3) Higher‐order themes based on the subthemes were extracted (Table 2). (4) The statistically significance of these themes and subthemes was interpreted and examined, and the data were revisited with these findings in consideration.

**TABLE 2:** Participant characteristics

Patient no	Age	Sex	Underlying disease	Aetiology	Wound site	Transfer	Treatment days	Type of device
A	70	F	Diabetic	Diabetic ulcer	Left mamma	Ambulatory	19	V.A.C. ULTA™^a^
B	79	F	Brain infarction, hyperlipidaemia	Surgical site infection	Precordium	Ambulatory	86	V.A.C. ULTA™
C	54	M	Hypertonia, hyperlipidaemia	Surgical site infection	Precordium	Ambulatory	89	V.A.C. ULTA™, V.A.C.®^b^
D	83	M	Hypertonia, hyperlipidaemia	Surgical site infection	Precordium	Ambulatory	83	V.A.C. ULTA™, V.A.C.®
E	67	M	Hypertonia	Necrotizing fasciitis	Precordium, femurs	Ambulatory	21	V.A.C. ULTA™
F	91	F		Ischemic skin ulcer	Right lower leg	Ambulatory	17	V.A.C. ULTA™
G	47	F		Traumatic ulcer	Right foot	Wheelchairs	5	V.A.C. ULTA™
H	42	M		Traumatic ulcer	Left foot	Wheelchairs	14	V.A.C. ULTA™
I	76	M	Brain infarction	Surgical site infection	Right groin	Ambulatory	20	V.A.C. ULTA™
J	45	M	Angina pectoris	Ulcer after tumour excision	Right buttock, femurs	Ambulatory	17	V.A.C. ULTA™, V.A.C.®
K	85	F	Angina pectoris, hypertonia	Surgical site infection	Right groin	Ambulatory	28	V.A.C. ULTA™
L	82	F	Hypertonia, hyperlipidaemia	Traumatic ulcer	Left finger	Ambulatory	18	V.A.C. ULTA™
M	43	M	Hypertonia, hyperlipidaemia	Traumatic ulcer	Right arm	Wheelchairs	10	V.A.C. ULTA™, V.A.C.®
N	26	M		Traumatic ulcer	Right finger	Ambulatory	8	V.A.C. ULTA™, V.A.C.®
O	78	M		Surgical site infection	Abdomen	Wheelchairs	15	V.A.C. ULTA™
P	83	F	Hypertonia, hyperlipidaemia	Ulcer after cicatrectomy	Right lower leg	Ambulatory	16	V.A.C. ULTA™
Q	52	M	Infant burn	Traumatic ulcer	Left arm	Ambulatory	17	V.A.C. ULTA™
Median	67						17	

Abbreviations: F, Female; M, Male.

^a^ V.A.C.ULTA™ Therapy System.

^b^ V.A.C.® Therapy System.

The data were coded by the first and third author. The co‐authors' opinions were then sought to validate the coding. All co‐authors specialized and were certified in wound, ostomy, continence nursing and plastic surgery. Group discussions were conducted to extract themes and subthemes. Following the discussions, an iterative process was completed among the authors to ensure clarity and confirm the accuracy of the analytical process, and the analytical results were described and shown to the participants. Next, a translator experienced in qualitative research translated the data. All authors confirmed the translated themes and subthemes. Post‐translation, the final agreement of the expert panel committee was confirmed.

### 2.5 Ethical considerations

Ethical approval was obtained from the relevant ethical review committee. This study was conducted in accordance with the ethical principles of human medical research set by the Declaration of Helsinki and all its amendments. All participants received oral and written information on the nature and purpose of the study before their participation. Participants were assured that participation withdrawal would not alter the type of care they would receive. All participants provided their written consent before inclusion in the study, and verbal consent was reconfirmed during the interview process. To ensure anonymity, participants were identified by pseudonyms during both interviews and transcripts.

## 3 RESULTS

The analysis included 17 participants (10 men and seven women), ranging from 26–91 years (*M*_age_ = 67). More than half of the patients were older than 65 years and were unemployed. Interviews lasted from 8–28 min (*M*_time_ = 16). Table 2 shows the participants and their characteristics.

Most participants had wounds caused by surgical site infections (SSIs) or trauma, and trunk was the most common region. At the time of the interviews, four participants were using wheelchairs. The number of NPWT treatment days ranged from 5–89 days (*M*_days_ = 17). All the participants used the V.A.C.ULTA™ Therapy System (KCI Inc.). Among 17 participants, five received sequential treatment by V.A.C.ULTA™ and the conventional V.A.C.® Therapy System (KCI Inc.).

Inpatients' themes related to their experience of NPWT treatment were characterized by the following seven themes: (1) pain and discomfort associated with treatment, (2) physical limitations owing to the attached device, (3) mental burden owing to the odour and noises of the attached device, (4) social limitations owing to the attached device, (5) advances in medical care and science, (6) device personification and (7) mixed feelings towards medical staff, each with its own subthemes. Table 3 shows the themes and subthemes of the patients' experiences throughout the NPWT.

**TABLE 3:** Patient experience during negative pressure wound therapy

Theme	Subtheme
1. Pain and discomfort associated with treatment	Having pain or discomfort as if one is being squeezed tightly There is strong pain when changing the foam dressing Treatment with continuous suction is more painful than that with continuous negative pressure and irrigation The pain is under control
2. Physical limitations owing to the attached device	Plugging in and unplugging the power cord when using the toilet is complicated Concerns about having problems with the tube while moving Inconvenience in moving or taking a shower
3. Mental burden owing to the odour and noises of the attached device	Concern over wound drainage, odour and device noise disturbing others in the surroundings Although the device sound is bothersome, it does not impede sleep Having to carry the device around all the time is stressful Awareness over the attached device hinders sleep Ability to put up with a little inconvenience if it meant healing the wound
4. Social limitations owing to the attached device	Impatience and anxiety about work and finances Mobility advantages of the attached device and the disadvantages of not being able to go outside
5. Advances in medical care and science	Feeling surprised by the wound healing acceleration effect of the new treatment method Feeling the advancement of medical care
6. Device personification	Feeling attachment towards the device Appreciation towards the device because it cleans the wound
7. Mixed feelings towards medical staff	There was little to no anxiety because the doctors explained the treatment method Because I trust the doctor, I leave the treatment to the professional Frustration towards medical staff who are unfamiliar with the device

### 3.1 Pain and discomfort associated with treatment

Twelve of the 17 participants experienced some pain or discomfort (i.e. a tightened feeling) after the treatment began. The degree of pain varied for every patient with six of 17 participants unable to tolerate pain without medication, whereas others experienced discomfort and less pain. Participants who reported feeling pronounced pain also reported that it worsened when changing the foam dressing. The five participants who were treated by both V.A.C.® and V.A.C.ULTA™ reported that V.A.C.ULTA™ was less painful than V.A.C.®. The following are excerpts of specific themes in patients' reports:

#### 3.1.1 Constant pain by being squeezed tightly

I experienced a pain of about NRS [numeric rating scale] 8/10 immediately after having the device applied for the first time. From the second day onward, I got used to the pain and had a continuous pain at about level 4. H: traumatic ulcer, 40s, male

I felt as if the wound was being sucked intensely. B: surgical site infection, 70s, female

I felt pain as if the wound/skin was being pulled. J: ulcer after tumour excision, 40s, male

#### 3.1.2 Pain when exchanging the foam dressing

The first time the foam dressing was changed, it was so painful that I had to be anesthetized to remove the foam dressing. M: traumatic ulcer, 40s, male

I felt a tingling because there was some pressure being applied to the wound. C: surgical site infection, 50s, male

#### 3.1.3 Pain from constant negative pressure being applied

Compared with the device that washes the wound, the device that only sucks the wound and the exudate sucks powerfully, thus it was extremely painful. M: traumatic ulcer, 40s, male

The device that only sucked the wound and the exudate was more painful than the device that washed the wound, and the pain was stronger than that of an open wound. N: traumatic ulcer, 20s, male

#### 3.1.4 The pain is under control

Because I took painkillers for 2 days after I had attached the device, I did not feel pain, but from the third day onward, I did not feel pain even when I didn't take painkillers. When the exudate in the wound was flushed, I said a flowing sensation, but it was not an unpleasant feeling. O: surgical site infection, 70s, male

I felt a little uncomfortable, but I didn't feel any pain or itchiness. I: surgical site infection, 70s, male

I didn't feel pain anywhere, and I thought it was really strange that the wound would heal with this. A: diabetic ulcer, 70s, female

### 3.2 Physical limitations owing to the attached device

Patients whose wounds were confined to their torsos said that the presence of a device or tube did not affect their movements and activities (e.g. showering). However, patients with wounds on their lower limbs, elder patients (aged 75 or over) and patients in wheelchairs were concerned about the power cords that had to be unplugged/plugged when using the toilet. Such patients were also concerned with possible complications with the tubes owing to moving. The following excerpts exemplify the statements above:

#### 3.2.1 Plugging in and unplugging the power cord when using the toilet is complicated

Because I am in a wheelchair, it was difficult to plug the cord in and out of the socket, unless the device was on top of the IV stand when I was moving to the bed. H: traumatic ulcer, 40s, male

Because I would go to the bathroom three times at night, I didn't like having to plug and unplug the cord because it was too much work. K: surgical site infection, 80s, female

Concerns about having problems with the tube while moving.

I was worried that the tube would get caught here and there when I stood up or moved around. I had my tube made long so that it was easy for me to move. However, there were times when I would run over it with the tire of the IV stand if the tube was too long. Q: traumatic ulcer, 50s, male

Since my sphere of activity was wide, having many tubes would get in my way. I would run over the tube with the tire of the IV stand when I was walking, which was troublesome to repair. N: traumatic ulcer, 20s, male

There is no inconvenience when moving or taking a shower.

The direction of the bed has been changed to make it easier for me to move onto the bed. Because the tube was made longer [in its extension], I don't feel any inconvenience when moving. I: surgical site infection, 70s, male

I don't think there is any inconvenience at all because I can take a shower once every four days by removing the foam material and the device. E: necrotizing fasciitis, 60s, male

### 3.3 Mental burden owing to the odour and noises of the attached device

Six out of 17 patients reported that they could not sleep because the attached device itself bothered them; another six out of 17 patients found it stressful to always carry an IV stand along with a device; and seven out of 17 patients were able to sleep even if the device emitted noises. These various responses show that there were individual differences about patients' experiences with the device. Furthermore, although some patients had a high level of tolerance towards the drainage, the smell of the drainage and the device noise, they were also worried that it might bother other people in the vicinity. Patients also reported being forced to endure various inconveniences and concerns to heal their wounds. The following excerpts show the aforementioned phenomena experienced by the patients:

#### 3.3.1 Concern about wound drainage, odour and device noise disturbing others in the surroundings

I didn't like it because the canister had dirty drainage from the wound, and I was worried that people I would pass by in the corridor might see it and think it was dirty. K: surgical site infection, 80s, female

I really didn't like how the smell from the tube, the canister, and the liquid excreted from the wound smelled like urine. I was worried that other patients, especially my ward mates and nurses, might also smell it. The operating sound of the device is at a level that is not bothersome if you don't pay attention to it. However, I'm not sure what my ward mates thought about the sound during a quiet night. I was worried that the noise generated during irrigation could disturb my ward mates. Q: traumatic ulcer, 50's, male

#### 3.3.2 Although the device sound is bothersome, it does not impede sleep

The buzzing sound bothered me at first, but I got used to it on the second day. After all, it would keep ringing and you'd get used to it. H: traumatic ulcer, 40s, male

I was not unable to sleep because of the operating sound. Q: traumatic ulcer, 50s, male

#### 3.3.3 Having to carry the device around all the time is stressful

I think it's a little troublesome because I always had to walk around pushing the IV stand with an attached device. I: surgical site infection, 70s, male

Because having the device attached to me was stressful, I didn't want to be wearing it for a long time. E: necrotizing fasciitis, 60s, male

#### 3.3.4 Awareness over the attached device hinders sleep

I slept with the tube on my chest because I was worried that the tube might get caught up while I was sleeping. I was finally able to sleep in around the last two days. I couldn't sleep until then because there were so many tubes. L: traumatic ulcer, 80s, female

I suffered from lack of sleep owing to a feeling of restriction by which the tube was attached. M: traumatic ulcer, 40s, male

Patients put up with a little inconvenience if it meant healing the wound.

There was a little inconvenience, but I can't say I don't want to wear a device that saves my life. E: necrotizing fasciitis, 60s, male

Although there was some inconvenience, I put up with it because I can't do anything unless the device heals my wounds. H: traumatic ulcer, 40s, male

### 3.4 Social limitations owing to the attached device

Those who had jobs were worried about work absenteeism and wanted to heal their wounds as quickly as possible so they could return to their jobs. Despite feeling anxious for financial reasons, patients hospitalized for a long time had to come to terms with the situation. Some patients understood that the Japanese health insurance system required patients under treatment with NPWT to remain hospitalized during the process, but they still wished they could go out while continuing treatment. Below are some excerpts describing the feelings of the patients:

#### 3.4.1 Impatience and anxiety about work and finances

I'm currently off work. I have tax bills and have gone past the point of impatience; I'm feeling resigned to the current situation. C: surgical site infection, 50s, male, long‐term hospitalization

Because I'm worried about my work, I want my wound to get healed fast so that I can leave the hospital as soon as possible. G: traumatic ulcer, 40s, female

#### 3.4.2 Mobility advantages of the attached device and the disadvantages of not being able to go outside

I think it's convenient that it's rechargeable because it allows for movement. E: necrotizing fasciitis, 60s, male

I'm grateful that the charged battery lasts for four to five hours. It allows me to go anywhere for a while. I: surgical site infection, 70s, male

It's inconvenient that I can't go out because I can't have the device unattached [from the body] for a long time. P: ulcer after cicatrectomy, 90s, female

I wish I could easily take it off when I want to go out for a bit. A: diabetic ulcer, 70s, female

### 3.5 Advances in medical care and science

Among our sample, 13 patients reported they said the curative effects of the NPWT treatment. They said their surprise and the accompanying joy related to the rapid healing process. Furthermore, they noted that they were impressed by the new technology and said their respect for its developers. The following excerpts describe the feelings of the patients:

#### 3.5.1 Feeling surprised by the wound healing acceleration effect of the new treatment method

It seems that I've gotten much better after receiving NPWT treatment, so I'm happy. I: surgical site infection, 70s, female

The progress of the treatment was good, and if I had not worn the device, the next operation would have been further delayed. H: traumatic ulcer, 40s, male

I was surprised that the wound healed so quickly. J: ulcer after tumour excision, 40s, male

The wound got better about 2 weeks after I started wearing this device. A: diabetic ulcer, 70s, female

#### 3.5.2 Feeling the advancement of medical care

I felt that the technology was advanced, unlike what we had in the past. I thought that I would immediately receive stitches, but I was impressed to know there is such a wonderful device like this one. Q: traumatic ulcer, 50s, male

I think the person who invented this device, which deftly cleans the wound as if by rinsing it with water, is admirable. L: traumatic ulcer, 80s, female

### 3.6 Device personification

Among the elder patients, there were those who gave a nickname to the device or who used words that would usually be reserved for people when referring to it. Some reported that they thought the device was like a friend. These respondents also described their feelings of gratitude towards the device for cleaning and healing their wounds. The following excerpts demonstrate this:

#### 3.6.1 Device personification; feeling of attachment towards the device

In the past, the doctor came in the morning and evening to clean. Now, this device has taken the place of the doctor. That's why I call this device ‘Dr. Roomba’. D: surgical site infection, 80s, male

I take ‘him’ with me wherever I go, so I feel a bit relieved when we depart temporarily during my shower; however, because I've been taking him around for a long time now, I missed him during my shower. I: surgical site infection, 70s, female

I take him around, like a friend, every day. I: surgical site infection, 70s, female

#### 3.6.2 Appreciation towards the device because it cleans the wound

The device is like something that cleans the wound, and I feel grateful toward it. O: surgical site infection, 70s, male

I thought I'd have to cut off this finger if there wasn't a treatment method for treating this wound by washing it, so I'm glad I could keep it. L: traumatic ulcer, 80s, female

I think it's something that makes me happy because it cleans the wound well. F: ischemic skin ulcer, 90s, female

### 3.7 Mixed feelings towards medical staff

Most patients received an explanation from their doctors and had an understanding of the treatment, which meant that they had little to no anxiety towards it. However, although some patients (particularly among the elder) did not understand the details of the treatment, they trusted their doctors, thus the former left the treatment (and all matters that came with it) to the care of the latter. Conversely, patients who had already been receiving treatment for a long period of time tended to express frustration towards staff who were not familiar with the handling of the devices. Here are some excerpts describing the feelings of the patients:

#### 3.7.1 There was little to no anxiety because the doctors had explained the treatment method

In terms of having anxiety regarding the treatment, I don't have such anxiety because I've received an explanation. M: traumatic ulcer, 40s, male

After I attached the device on, I asked my doctor various questions since I was wondering what kind of treatment it was. The doctor then gave me a pamphlet with a simple description, and I understood what kind of treatment it was by reading the pamphlet. G: traumatic ulcer, 40s, female

#### 3.7.2 Because I trust the doctor, I leave the treatment to the professional

I leave the treatment up to my doctor because I believe that I'll be cured if I receive the given treatment. H: traumatic ulcer, 40s, male

No matter what I say, I have no knowledge of it, so I feel like just leaving it [up to the doctor]. B: surgical site infection, 70s, female

#### 3.7.3 Frustration towards medical staff who is unfamiliar with the device

I think the medical staff is hardly used to handling the device; like, the canister has not been replaced, even when it is filled with drainage. It really irritates me. It's been about 4–5 months since the start of the treatment. C: surgical site infection, 50s, male

## 4 DISCUSSION

This study aimed to clarify the experiences of receiving NPWT therapy. The most common themes were pain and discomfort associated with treatment, physical limitations owing to the attached device, mental burden owing to the odour and noises of the attached device, social limitations owing to the attached device, advances in medical care and science, device personification and mixed feelings towards medical staff.

### 4.1 Pain and discomfort associated with treatment

Pain and discomfort associated with treatment was the most salient theme in this study. Based on our clinical experience and other reports acknowledging pain associated with NPWT, the removal of the dressing can be, and usually is, a particularly painful experience for patients. This is often perceived as a side effect of NPWT therapy by patients and medical personnel (Christensen et al., 2013). For this reason, six out of the 17 patients in this study were already using analgesics when the study started. However, despite the active use of analgesics, many patients remained in pain. Abbotts (2010) reported that many people complained of pain due to the NPWT, whereas others did not, and said pain or comfort at various levels. Additionally, five out of 17 participants underwent both the V.A.C.® and the V.A.C.ULTA™ treatments, and all five stated that the V.A.C.ULTA™ treatment was less painful than that of the V.A.C.®. Medical staff should endeavour to alleviate changes in pain depending on the type of equipment used and while changing dressings.

### 4.2 Physical limitations and mental burden

About physical limitations owing to the attached device and the mental burden owing to the odour and noises of the attached device, previous studies on NPWT have highlighted patients' physical and social limitations. Patients reported to having to stay in bed because the tube is connected to the wound, the suction is continuously performed, and they preferred not to venture outdoors owing to the bad odour (Abbotts, 2010). Additionally, it was bothersome and cumbersome to access the toilet, manoeuvre the machine without tripping and unplug (Bolas & Holloway, 2012). These same limitations were highlighted in this study, which found that patients with physical limitations (wounds on their lower limbs, elder aged and in wheelchairs) connected to an NPWT device and were concerned about the power cords being unplugged or plugged, due to any movement. Many patients were also bothered by the attached device itself and the tubes, power cords, noise and smell from the device. Therefore, patients, particularly those with physical limitations, should be provided additional information about the falling risks due to the device, and about the need for care with it. Nurses need an increased amount of time to observe the patients in this study, most patients did not report due to the drainage odour and device noise; rather, they were concerned about its unpleasant nature for others. Such feelings may be described as Omoiyari (altruistic sensitivity), which is particular to Japanese patients (Hara, 2006). Therefore, medical workers need to ensure that the noises and alarms from the devices do not disturb other patients in the same room (e.g. covering the drainage in a way that it is not bothersome to other people). Further, although patients in the West were able to receive at‐home NPWT, and were thus, able to return to their social lives postdischarge, they experienced social limitations, such as being unable to wear fashionable clothes or shoes and being forced to resign from demanding jobs that require physical stamina (Abbotts, 2010; Bolas & Holloway, 2012). Thus, need to be given education about their self‐care. As NPWT remains an inpatient‐only treatment in Japan, the social limitations are evident. To help reduce these social limitations, policy‐makers should exert efforts for the inclusion of at‐home NPWT treatment in health insurance. Additionally, medical workers may need to adjust and consider the possibility of patients temporarily using portable NPWT devices if they feel the need to venture out.

### 4.3 Perception of the treatment of inpatients undergoing NPWT

About the inpatients' perceptions towards their NPWT treatment, the following three themes were derived: advances in medical care and science, device personification and mixed feelings towards medical staff.

Most patients (13 of 17 participants) in this study recognized that NPWT was an advance in medical care and science and said their surprise and accompanying joy related to the rapid healing process. Abbotts (2010) reported that improving disease is extremely important to patients, as it gives them hope and makes them interested in the treatment. Therefore, medical staff should explain to patients that NPWT can make their wounds heal rapidly, which can increase the patients' interest in and understanding of the treatment and give them hope for recovery. Such actions could make patients tolerate the various inconveniences mentioned above.

Device personification was the most characteristic theme in this study; that is, most patients reported having some type of personification towards the device. Patients not only perceived the devices as objects that brought negative impact but also tended to perceive their devices as being something positive. A previous study (Kino et al., 2006) showed that people tend to see something they frequently carry with themselves as a part or an extension of their bodies, and, at times, people may share their identities with such objects. Amid trying to accept the difficult situations they found themselves in, patients may start perceiving their devices positively owing to the healing of their wounds and empathizing with the devices rather than treating them as a nuisance.

Hence, in a nursing setting, nurses may incentive the patients to treat their attached devices as their companions or friends, which may lead to higher positive perceptions of the treatment.

About the patients' mixed feelings towards medical staff, many said frustrated with inexperienced medical workers. This finding indicated the need to provide education and training for nurses and corresponds with the previous research (Abbotts, 2010; Bolas & Holloway, 2012). As NPWT has gained wider adoption and interest partly due to the increasing complexity of wounds and patient conditions (Kim et al., 2020), the number of medical staff who interacted with patients treated with NPWT has also increased. It is important that staff receive education from professionals (wound, ostomy and continence nurses in Japan), to unify and improve their skills and ensure clear communication with patients.

## 5 CONCLUSION

Patients underwent NPWT in this study reported physical limitations about pain and device attachment, mental burdens related to odour and device noise, and social limitations related to not being able to return to work or go home. Although participants were concerned about disturbing others during their treatment, they also desired an improvement in their condition. Thus, the patients seemed to tolerate the aforementioned limitations while feeling attachment and gratitude towards the device and the medical staff for helping them heal.

However, this study had some limitations. As this study was conducted in a single facility with patients above 20 years of age and incapable of independently performing activities of daily living and the field was limited to the plastic surgery ward, its inherent limitation is the lack of generalizability to other populations.

## CONFLICT OF INTEREST

The authors declare no conflicts of interest.

## ETHICAL APPROVAL

The authors state that the procedures followed were in compliance with the regulations of the Clinical Research Ethics Committee and the World Medical Association and the Declaration of Helsinki. They declare that they have complied with their site's protocols for the publication of patient data and that all patients included in the study received sufficient information and provided their written informed consent to participate in this study.

## Data Availability

The data that support the findings of this study are available on request from the corresponding author. The data are not publicly available due to privacy or ethical restrictions.

## References

[nop21392-bib-0001] Abbotts, J. (2010). Patients' views on topical negative pressure: ‘Effective but smelly’. British Journal of Nursing, 19(20), S37–S41. 10.12968/bjon.2010.19.Sup10.79692 21072010

[nop21392-bib-0002] Andrews, A. , & Upton, D. (2013). Negative pressure wound therapy: Improving the patient experience part 3 of 3. Journal of Wound Care, 22(12), 671–672. 10.12968/jowc.2013.22.12.671 24335891

[nop21392-bib-0003] Bolas, N. , & Holloway, S. (2012). Negative pressure wound therapy: A study on patient perspectives. British Journal of Community Nursing, 17(Sup3), S30–S35. 10.12968/bjcn.2012.17.sup3.s30 22584182

[nop21392-bib-0004] Christensen, T. J. , Thorum, T. , & Kubiak, E. N. (2013). Lidocaine analgesia for removal of wound vacuum‐assisted closure dressings: A randomized double‐blinded placebo‐controlled trial. Journal of Orthopaedic Trauma, 27(2), 107–112. 10.1097/BOT.0b013e318251219c 23343829

[nop21392-bib-0005] Gray, J. R. , Grove, S. K. , & Sutherland, S. (2017). Burns and Grove's the practice of nursing research; Appraisal, synthesis, and generation of evidence (8th ed.). Elsevier.

[nop21392-bib-0006] Hara, K. (2006). The concept of Omoiyari (altruistic sensitivity) in Japanese relational communication. Intercultural Communication Studies, 15(1), 24–32.

[nop21392-bib-0007] Huang, C. , Leavitt, T. , Bayer, L. R. , & Orgill, D. P. (2014). Effect of negative pressure wound therapy on wound healing. Current Problems in Surgery, 51(7), 301–331. 10.1067/j.cpsurg.2014.04.001 24935079

[nop21392-bib-0008] Kim, P. J. , Attinger, C. E. , Constantine, T. , Crist, B. D. , Faust, E. , Hirche, C. R. , Lavery, L. A. , Messina, V. J. , Ohura, N. , Punch, L. J. , Wirth, G. A. , Younis, I. , & Téot, L. (2020). Negative pressure wound therapy with instillation: International consensus guidelines update. International Wound Journal, 17(1), 174–186. 10.1111/iwj.13254 31667978 PMC7003930

[nop21392-bib-0009] Kino, K. , Iwaki, T. , Ishihara, S. , & Dekihara, H. (2006). An analysis of the attachment to artifacts: A study using the analogy of interpersonal relationships. Journal of Japan Society of Kansei Engineering, 6(2), 33–38. 10.5057/jjske2001.6.2_33

[nop21392-bib-0010] Liamputtong, P. , & Ezzy, D. (2005). Qualitative research methods (2nd ed.). Oxford University Press.

[nop21392-bib-0011] Moffatt, C. J. , Mapplebeck, L. , Murray, S. , & Morgan, P. A. (2011). The experience of patients with complex wounds and the use of NPWT in a home‐care setting. Journal of Wound Care, 20(11), 512–527. 10.12968/jowc.2011.20.11.512 22240846

[nop21392-bib-0012] Morykwas, M. J. , Argenta, L. C. , Shelton‐Brown, E. I. , & McGuirt, W. (1997). Vacuum‐assisted closure: A new method for wound control and treatment: Animal studies and basic foundation. Annals of Plastic Surgery, 38(6), 553–562. 10.1097/00000637-199706000-00001 9188970

[nop21392-bib-0013] Neubauer, B. E. , Witkop, C. T. , & Varpio, L. (2019). How phenomenology can help us learn from the experiences of others. Perspectives on Medical Education, 8, 90–97. 10.1007/s40037-019-0509-2 30953335 PMC6468135

[nop21392-bib-0014] Ottosen, B. , & Pedersen, B. D. (2013). Patients' experiences of NPWT in an outpatient setting in Denmark. Journal of Wound Care, 22(4), 197–206. 10.12968/jowc.2013.22.4.197 23702673

[nop21392-bib-0015] Polit, D. F. , & Beck, C. T. (2017). Nursing research: Generating and assessing evidence for nursing practice (10th ed.). Wolters Kluwer.

[nop21392-bib-0016] Seidman, I. (2006). Interviewing as qualitative research: A guide for researchers in education and the social sciences. Teachers College Press.

[nop21392-bib-0017] Upton, D. , & Andrews, A. (2013). Negative pressure wound therapy: Improving the patient experience part 3 of 2. Journal of Wound Care, 22(12), 584–591. 10.12968/jowc.2013.22.11.582 24335891

[nop21392-bib-0018] Upton, D. , & Andrews, A. (2015). Pain and trauma in negative pressure wound therapy: A review. International Wound Journal, 12(1), 100–105. 10.1111/iwj.12059 23489350 PMC7950877

[nop21392-bib-0019] Zens, Y. , Barth, M. , Bucher, H. C. , Dreck, K. , Felsch, M. , Groß, W. , Jaschinski, T. , Kölsch, H. , Kromp, M. , Overesch, I. , Sauerland, S. , & Gregor, S. (2020). Negative pressure wound therapy in patients with wounds healing by secondary intention: A systematic review and meta‐analysis of randomized controlled trials. Systematic Reviews, 9(1), 238. 10.1186/s13643-020-01476-6 33038929 PMC7548038

